# Supplementary material for: External Validation of COVID-19 Risk Scores during Three Waves of Pandemic in a German Cohort—A Retrospective Study
Source: J Pers Med. 2022 Oct 28;12(11):1775. doi: 10.3390/jpm12111775 (PMC9693591; doi:10.3390/jpm12111775)
Supplement: Supplementary file 1 [file jpm-12-01775-s001.zip › jpm-1955855-supplementary.pdf]

Supplementary

Table S1 Missing variables in calculation of scores for N=347

Missing Score variable	Score	First cohort N (%)	Second cohort N (%)	Third cohort N (%)
Urea	4C-Score	33 (24.6)	91 (48.7)	22 (84.6)
Bilirubine	COVID-GRAM	11 (8.2)	42 (22.5)	7 (26.9)
LDH	COVID-GRAM	13 (9.7)	39 (20.9)	7 (26.9)
Respiratory rate	4C-Score, qSOFA, CRB-65, NEWS	10 (7.5)	36 (19.3)	5 (19.2)
Oxygen supply	NEWS	10 (7.5)	27 (14.4)	5 (19.2)
Oxygen saturation	4C-Score, NEWS	10 (7.5)	27 (14.4)	5 (19.2)
Temperature	NEWS	9 (6.7)	26 (13.9)	5 (19.2)
Systolic blood pressure	CRB-65, qSOFA, NEWS	8 (6.0)	25 (13.4)	5 (19.2)
Heart rate	NEWS	8 (6.0)	24 (12.8)	5 (19.2)
GCS	4C-Score, CRB-65, qSOFA, COVID-GRAM, NEWS	8 (6.0)	24 (12.8)	5 (19.2)
CRP	4C-Score	7 (5.2)	18 (9.6)	3 (11.5)

Table S1. Missing variables in calculation of scores at patients admission for N=347 patients of study population. Abbreviations: *COVID* Coronavirus Disease, *GRAM* Guangzhou Institute of Respiratory Health Calculator at Admission, *qSOFA* Quick Sequential Organ Failure Assessment, *CRB* Confusion, Respiratory rate, Blood pressure, *NEWS* National Early Warning Score, *LDH* Lactate dehydrogenase, GCS Glasgow Coma Scale, *CRP* C-reactive protein

Table S2 Characteristics of excluded patients

	CRB-65 N (% or Q1-Q3)	NEWS N (% or Q1-Q3)	4C-Score N (% or Q1-Q3)	COVID-GRAM N (% or Q1-Q3)	qSOFA N (% or Q1-Q3)
Overall	51	66	194	98	51
Female	26 (51.0)	35 (53.0)	95 (49.0)	55 (56.1)	26 (51.0)
Average age	59.3 (46.0-76.0)	61.2 (50.0-76.0)	62.9 (53.0-76.0)	65.9 (59.0-80.0)	59.3 (46.0-76.0)
ICU admission	10 (19.6)	17 (25.8)	25 (12.9)	16 (16.3)	10 (19.6)
In-hospital mortality	3 (5.9)	6 (9.1)	14 (7.2)	8 (8.2)	3 (5.9)
Cohort 1	10 (19.6)	17 (25.8)	47 (24.2)	20 (20.4)	10 (19.6)
Female	4 (40.0)	10 (58.8)	22 (46.8)	13 (65.0)	4 (40.0)
Average age	52.8 (33.8-80.0)	56.1 (46.0-74.0)	62.7 (50.0-80.0)	64.9 (50.8-80.8)	52.8 (33.8-80.0)
ICU admission	5 (50.0)	7 (41.2)	14 (29.8)	8 (40.0)	5 (50.0)
In-hospital mortality	2 (20.0)	3 (17.6)	10 (21.3)	4 (20.0)	2 (20.0)
Cohort 2	36 (70.6)	44 (66.7)	124 (63.9)	66 (67.3)	36 (70.6)
Female	20 (55.6)	23 (52.3)	63 (50.8)	36 (54.5)	20 (55.6)
Average age	61.9 (55.0-76.0)	63.9 (56.0-76.0)	63.8 (55.0-76.0)	67.4 (60.0-81.0)	61.9 (55.0-76.0)
ICU admission	4 (11.1)	9 (20.5)	10 (8.1)	7 (10.6)	4 (11.1)
In-hospital mortality	1 (2.8)	3 (6.8)	4 (3.2)	4 (6.1)	1 (2.8)
Cohort 3	5 (9.8)	5 (7.6)	23 (11.9)	12 (12.2)	5 (9.8)
Female	2 (40.0)	2 (40.0)	10 (43.5)	6 (50.0)	2 (40.0)
Average age	53.8 (36.0-71.0)	53.8 (36.0-71.0)	58.5 (48.0-68.0)	59.5 (49.8-68.0)	53.8 (36.0-71.0)
ICU admission	1 (20.0)	1 (20.0)	1 (4.3)	1 (8.3)	1 (20.0)
In-hospital mortality	0 (0)	0 (0)	0 (0)	0 (0)	0 (0)

Table S2 Characteristics of excluded patients for each score studied. Abbreviations: *CRB* Confusion, Respiratory rate, Blood pressure, *NEWS* National Early Warning Score, *COVID* Coronavirus Disease, *GRAM* Guangzhou Institute of Respiratory Health Calculator at Admission, *qSOFA* Quick Sequential Organ Failure Assessment, *ICU* Intensive Care Unit

Table S3 Discriminatory indices at several cut-offs of the scores[1]

	Cut-off	Prevalence (95% CI)	Sensitivity (95% CI)	Specificity (95% CI)	Positive predictive value (95% CI)	Negative predictive value (95% CI)
**ICU-admission**						
qSOFA	≥2	24.3 (19.5-29.3)	15.3 (7.5-24.2)	98.2 (96.3-99.6)	73.4 (50.0-93.8)	78.3 (73.4-83.1)
COVID-GRAM[2]	≥1.7%	-	-	-	-	-
	≥40.4%	26.5 (21.0-32.0)	98.5 (94.9-100)	3.8 (1.2-6.9)	27.0 (21.4-32.6)	87.5 (57.1-100)
NEWS	≥5	22.9 (18.1-27.9)	89.0 (80.7-96.2)	64.1 (57.7-70.4)	42.5 (34.2-51.0)	95.2 (91.3-98.5
	≥7	22.9 (18.1-27.9)	67.1 (55.4-78.3)	81.8 (76.5-86.9)	52.4 (41.5-63.3)	89.3 (84.7-93.4)
CRB-65	≥2	24.3 (19.5-29.3)	44.4 (32.8-56.2)	87.5 (82.9-91.6)	53.3 (40.4-66.1)	83.0 (78.2-87.7)
4C-Score	≥4	37.2 (29.6-45.0)	98.2 (94.0-100)	2.1 (0-5.4)	37.3 (29.6-45.2)	66.5 (0-100)
	≥9	37.2 (29.6-45.0)	93.0 (85.7-98.4)	22.9 (14.8-31.6)	41.7 (33.1-50.4)	84.6 (69.6-96.6)
	≥15	37.2 (29.6-45.0)	21.0 (10.9-32.0)	96.9 (92.9-100)	79.9 (57.1-100)	67.4 (59.4-75.0)
**In-hospital mortality**						
qSOFA	≥2	9.8 (6.5-13.3)	6.9 (0-17.6)	95.1 (92.4-97.5)	13.3 (0-33.3)	90.4 (86.8-93.7)
COVID-GRAM[2]	≥1.7%	-	-	-	-	-
	≥40.4%	9.6 (6.1-13.5)	100	3.6 (1.3-6.2)	9.9 (6.3-13.9)	100
NEWS	≥5	9.3 (6.0-12.9)	84.6 (69.2-96.7)	55.7 (49.6-61.7)	16.4 (10.4-23.0)	97.2 (94.3-99.4)
	≥7	9.3 (6.0-12.9)	69.2 (50.0-86.4)	74.7 (69.2-80.0)	21.9 (13.3-31.4)	95.9 (93.0-98.5)
CRB-65	≥2	9.8 (6.5-13.3)	48.3 (30.0-66.7)	82.7 (78.1-87.1)	23.3 (13.0-34.5)	93.6 (90.4-96.6)
4C-Score	≥4	11.8 (6.9-17.1)	100	2.2 (0-5.1)	12.0 (7.1-17.5)	100
	≥9	11.8 (6.9-17.1)	100	19.3 (12.8-26.1)	14.2 (8.5-20.6)	100
	≥15	11.8 (6.9-17.1)	33.3 (11.8-57.1)	93.3 (88.8-97.2)	40.1 (14.3-66.7)	91.3 (86.3-95.7)

[1]Score cut-offs according to derivation literature
[2]COVID-GRAM risk of critical illness. We only assessed discriminatory indices at the higher cut-off because we had no patient with a score value less than 1.7%

Table S3 Discriminatory indices at several clinical cut-offs of the scores (defined by international literature) regarding the overall study population. Abbreviations: *ICU* Intensive Care Unit, *qSOFA* Quick Sequential Organ Failure Assessment, *COVID* Coronavirus Disease, *GRAM* Guangzhou Institute of Respiratory Health Calculator at Admission, *NEWS* National Early Warning Score, *CRB* Confusion, Respiratory rate, Blood pressure

Figure S1

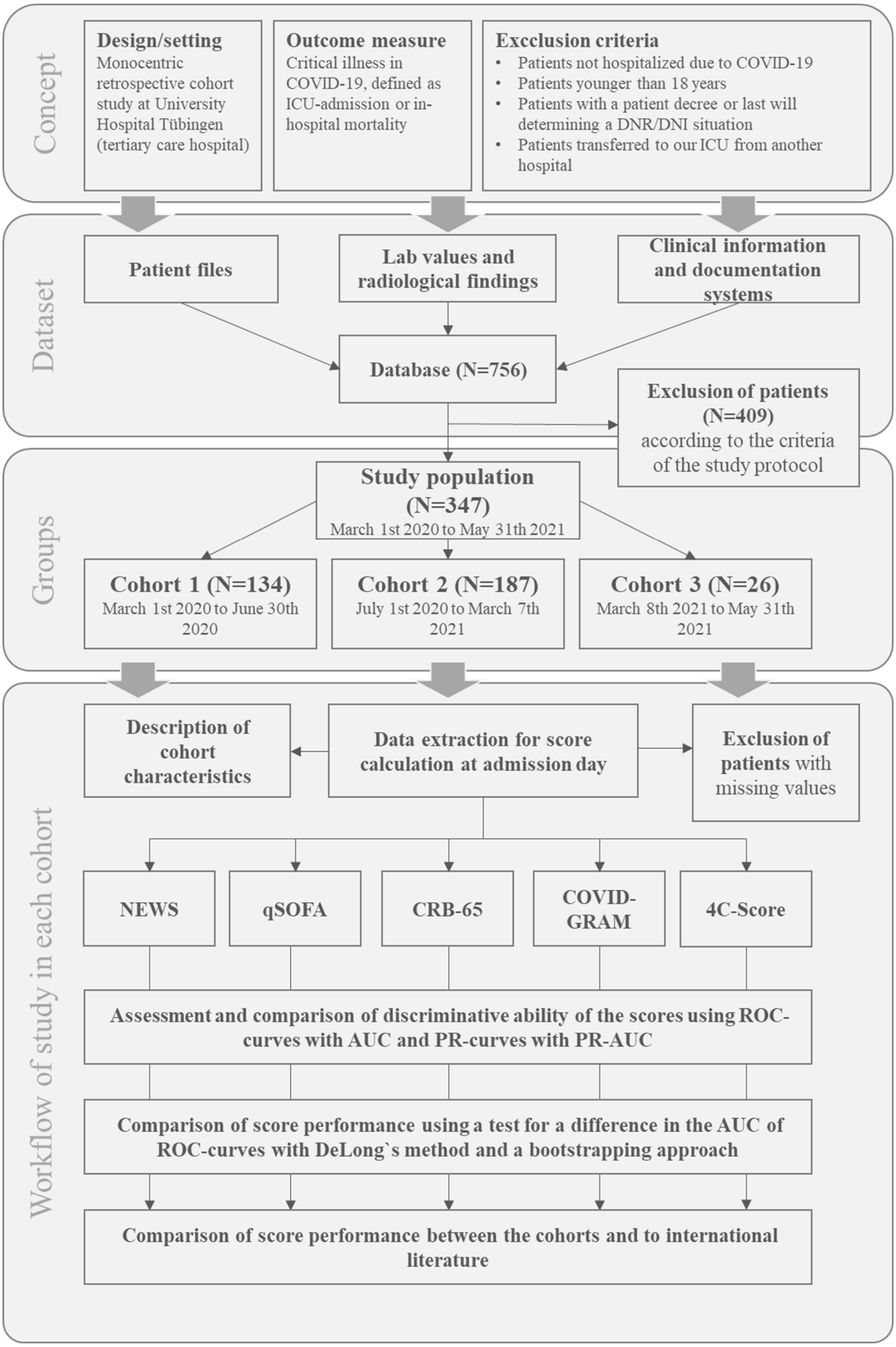

Fig S1. Schematic workflow of study. Study concept (upper panel), dataset and groups (middle panel), the workflow of score calculation, validation, and comparison in each cohort (lower panel). Abbreviations: *COVID* Coronavirus Disease, *ICU* Intensive Care Unit, *DNR* Do not intubate, *DN*I Do not resuscitate, *CRB* Confusion,

Respiratory rate, Blood pressure, *NEWS* National Early Warning Score, *GRAM* Guangzhou Institute of Respiratory Health Calculator at Admission, *qSOFA* Quick Sequential Organ Failure Assessment, *AUC* Area under the curve, *PR* Precision recall, *ROC* Receiver Operating Characteristics

Figure S2

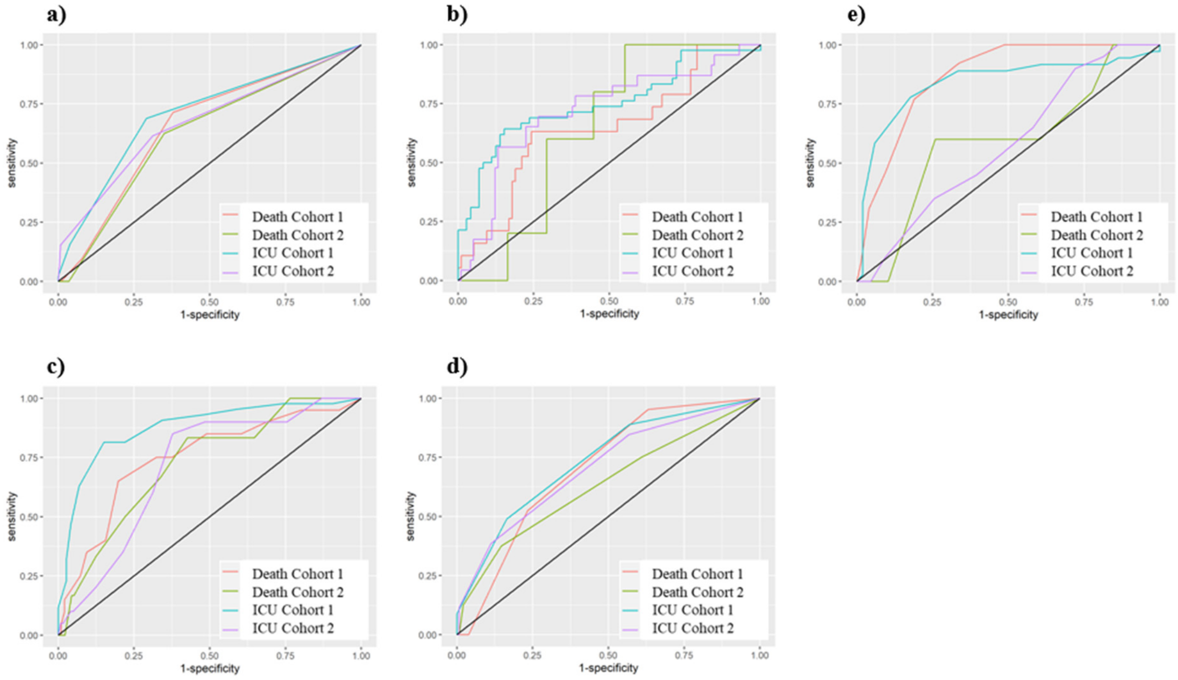

Figure S2. Plots of Area under the receiver operating characteristics of qSOFA (a), COVID-GRAM (b), NEWS (c), CRB-65 (d) and 4C-Score (e) with regard to ICU admission and in-hospital mortality in the first and the second cohort. Abbreviations: *ICU* Intensive Care Unit
